# A comparison of U.S. infant feeding policy changes to Global Breastfeeding Collective policy priorities

**DOI:** 10.3389/fpubh.2025.1653377

**Published:** 2025-09-17

**Authors:** Paige B. Harrigan, Todd Schenk, Stella L. Volpe, Valisa E. Hedrick, Tuba Khan, Sarah A. Misyak

**Affiliations:** ^1^Department of Human Nutrition, Foods, and Exercise, Virginia Polytechnic Institute and State University (Virginia Tech), Blacksburg, VA, United States; ^2^School of Public and International Affairs, Virginia Tech, Blacksburg, VA, United States; ^3^Department of Nutritional Sciences, Oklahoma State University, Stillwater, OK, United States

**Keywords:** breastfeeding, Global Breastfeeding Collective, infant feeding, infant formula, policy

## Abstract

**Introduction:**

Public policy plays an important role in shaping how infants are fed. The Global Breastfeeding Collective (GBC) provides a set of policy priorities for countries to promote, protect and support breastfeeding. The GBC uses scorecards to document progress toward meeting those priorities. The purpose of this study was to assess recent United States (U.S.) federal infant feeding policy changes against the GBC's policy priorities to identify areas of alignment and gaps for policies supporting optimal infant feeding.

**Methods:**

Changes in U.S. federal infant feeding legislation, regulation, and presidential documents between 2014 and 2023 were compared with and coded into GBC priority categories. Policy changes not aligned with GBC priorities were coded into additional non-GBC topic categories that were developed inductively.

**Results:**

Fifty-seven federal infant feeding policies were adopted or substantively modified within the study period. Of these, only 17 aligned with at least one of the GBC policy priorities. Forty-nine policies included changes that did not match GBC policy priorities. Policy changes that did not align with GBC priorities addressed infant formula manufacturing, lactation spaces, and breastfeeding supplies, among other topics.

**Conclusion:**

Although most recent federal infant feeding policy changes in the U.S. did not align with the breastfeeding policy priorities established by the GBC, opportunities to promote and protect breastfeeding were identified. Some U.S. breastfeeding policy changes outside of GBC priorities have potential to strengthen breastfeeding.

## 1 Introduction

Despite known benefits, breastfeeding rates in the United States (U.S.) fall short of the U.S. Government's Healthy People 2030 objectives, which include increasing the percent of infants exclusively breastfed to at least six months of age to 42.4% and increasing the percentage of infants who are breastfed at 1 year to at least 54.1% (1–3). In 2025, U.S. rates are 27.7% for exclusive breastfeeding to 6 months and 39.5% for continued breastfeeding through 12 months (4). Factors contributing to the U.S. not meeting breastfeeding objectives include the effects of unrestricted infant formula marketing, the absence of comprehensive paid maternity leave and workplace lactation policies, and social norms that do not encourage breastfeeding, which are underscored by ethnic, racial, income, and educational disparities (3, 5–7).

Public health nutrition experts endorse public policies that support and promote optimal feeding of infants and young children (0–24 months) in the U.S. and globally (5, 8–10). Increasing breastfeeding behaviors would contribute to reaching both Healthy People 2030 national public health aims in the U.S. and the United Nation's Sustainable Development Goals (SDGs) (11, 12). The SDGs are a set of global priorities to improve health and education, increase economic growth, and reduce inequalities.

In 2017, the Global Breastfeeding Collective (GBC), a global partnership of agencies, including the US Agency for International Development and the U.S. Centers for Disease Control and Prevention (CDC), was launched by the World Health Organization (WHO) and the United Nations Children's Fund to speed global progress toward the achievement of breastfeeding targets by coalescing around shared policy priorities in the WHO's Global Strategy for Infant and Young Child Feeding (10, 13–15). The aim of the GBC is to increase the adoption, implementation, and enforcement of recommended policy priorities that protect, promote, and support breastfeeding. The GBC calls on governments to (1) increase funding to raise breastfeeding rates; (2) fully implement the International Code for Marketing of Breastmilk Substitutes (“the Code”), a set of legal guidelines aimed to restrict the promotion of infant formula by industry to protect breastfeeding, which the U.S. has not formally adopted (16–18); (3) enact paid family leave and workplace policies; (4) implement the “Ten Steps to Successful Breastfeeding” in maternity facilities, a package of health care programs and policies in support of breastfeeding and to follow the Code in facilities designated as Baby-Friendly (19, 20); (5) improve access to skilled breastfeeding counseling; (6) strengthen links between health facilities and communities; (7) strengthen monitoring systems that track the progress of [breastfeeding] policies, programs and funding; and [expand]; (8) infant and young child support in emergencies (13). These policy priorities are recommended for all countries, developed and developing.

The GBC uses data sources, including from the CDC and the World Breastfeeding Trends Initiative (WBT*i*), to track country-level implementation and performance across recommended policy priorities to monitor and assess progress toward the targets in the Global Breastfeeding Scorecard (10, 21–23). The WBT*i*, which is a collaborative network, follows country-level progress toward global targets to assess, monitor, and publicly display infant and young child feeding programs and policy progress using standardized metrics, and it compiles country reports that are used to update the GBC Scorecards (21–24). Based on 2019 data, the most recently available, the U.S. scored 40.5 on a 100-point scale, ranking it in the bottom quintile of countries, underscoring the need for improvements (15, 25).

Most U.S. infants are fed infant formula in the first year of life for part or all of their nutrients and infant diets globally are moving to formula feeding (26, 27). For families feeding infants with infant formula, it is an essential food (28). The COVID-19 pandemic and 2022 infant formula shortage (henceforth, “shortage”) in the U.S. affected infant formula supply chains, shifted breastfeeding and formula feeding practices, and affirmed infant formula as a critical food source to be protected (28–31). The shortage highlighted systematic inequalities, as populations with low incomes and from racial and ethnic minority groups were more likely to have to take on negative coping behaviors in response to the shortage (32–34). In light of the shortage, policymakers called for action to maintain and protect infant formula supply and public health nutrition experts urged policy responses that would support breastfeeding, in addition to protecting infant formula supply over the long-term (28, 29, 32, 35, 36).

The purpose of this study was to determine how federal U.S. infant feeding policy changes compare to the GBC policy area priorities. The extent to which U.S. federal policies, which tend to be stable and resistant to change (37), were adopted and substantively modified indicates attention to infant feeding as a public policy issue (38). A comparison of policy changes to GBC policy priorities identified through a policy scan assessing the timing and content of changes can be used to identify priorities and gaps in U.S. infant feeding policies and opportunities to enhance U.S. infant feeding policies within the highly connected and rapidly changing global food system (14, 39, 40).

## 2 Materials and methods

### 2.1 Study design

This study was part of a larger policy scan of U.S. infant feeding policy changes (41). Three online government policy databases—USCODE.house.gov, eCFR.gov, and federalregister.gov—and one legal research database, Westlaw Edge, were used to identify changes in federal infant feeding legislation, regulation, and presidential document policies between January 1, 2014 and December 31, 2023 (42–45). Federal level legislative, regulatory and executive policies were selected because they are visible reflections of policymaker attention (46). An example of a legislative policy is 42 USC §1786, the law that covers the Special Supplemental Nutrition Program for Women, Infants, and Children (WIC). A regulatory policy example is 21 CFR §106.91, which regulates infant formula quality control processes. An example of a presidential document is 79 FR 36625, which is a presidential memorandum entitled Enhancing Workplace Flexibilities and Work-Life Programs that enhances support for lactation in workplaces. Presidential documents refer to policies that are issued to guide the executive branch of government, including executive orders and memorandums. Policy changes over a 10-year period were included in the study to provide an adequate timespan to compare changes over time and is consistent with other public health nutrition policy scans (47, 48).

The study protocol, which was adapted from policy scanning processes used in previous research, involved (1) searching, identifying and screening infant feeding policies followed by reviewing verifying and extracting policy changes for inclusion in the study; and (2) analyzing, interpreting, and comparing relevant infant feeding policy changes (49–51), against recommended GBC breastfeeding policy priorities. The analysis followed seven policy area recommendations prioritized by the GBC (Table 1). The GBC recommendation to “Increase funding to raise breastfeeding rates from birth to 2-years” was outside of the scope of the analysis of infant feeding policies and thus not included, because funding levels are determined through separate appropriation procedures, not within the policy itself.

**Table 1:** Definitions for categorization of U.S. federal infant feeding policy changes according to Global Breastfeeding Collective (GBC) policy priorities in a U.S. context.

**GBC policy priority**	**Definition**
Fully implement the Code of Marketing of Breastmilk Substitutes	Policy relates to how infant formula would be advertised or marketed. Given that the US has not signed onto the Code, it was not expected that GBC policy aims specific to enacting the Code into legislation or Code enforcement would be identified and therefore a broader scope of around advertisement or marketing.
Enact paid family leave and workplace policies	Any family leave and/or workplace breastfeeding policies. Workplace policies could refer to breaktimes, and/or anti-discrimination policies related to lactation. Lactation spaces at work were coded into this GBC category. Lactation spaces in public in a separate category.
Implement the Ten Steps to Successful Breastfeeding in maternity facilities	Any policy describing facility level infant feeding in hospitals including, but not limited to, Baby-Friendly Hospitals.
Improve access to skilled breastfeeding counseling	Any policy including training and skill development for lactation counselors, in alignment with the GBC Inclusion of IYCF Support in Pre-Service Curricula indicator.
Strengthen links between health facilities and communities	Policy relates to community level breastfeeding support programs or networks that increase access to lactation counseling and/or referral to counseling.
Strengthen monitoring systems that track the progress of [breastfeeding] policies, programs, and funding	Policy covers the monitoring or tracking of breastfeeding, whether in policies, programs, or funding. Research in a separate category.
Infant and Young Child Feeding in Emergencies	Policy describes the inclusion or expansion infant feeding support in humanitarian, emergency, or disaster contexts, in the US Also refers to US infant feeding support in foreign assistance funding.

Sources: Global Breastfeeding Collective Seven Policy Actions (2020) (13), GBC Scorecard (2023) (21), GBC Indicators for the Seven Policy Priorities Interactive Dashboard (2024) (23).

### 2.2 Policy screening, verification, extraction

**Figure 1:**
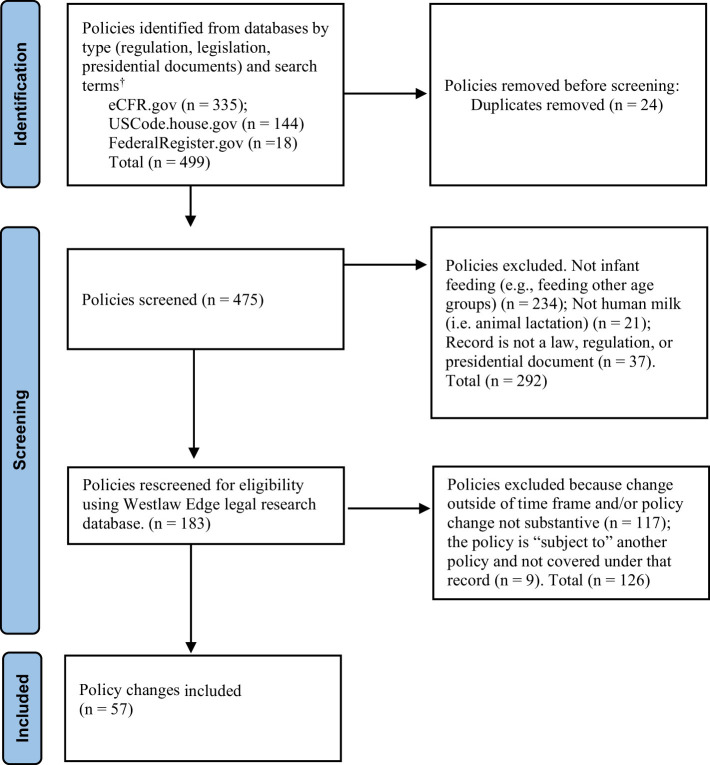
PRISMA style workflow diagram for identification of U.S. infant feeding policy changes from 2014 through 2023.^†^Search terms by regulations; breast* (*n* = 189); lactation (*n* = 32); “infant formula” (*n* = 105); “baby formula” (*n* = 1); “infant feeding” (*n* = 7); “infant nutrition” (*n* = 1); legislations; breast*(*n* = 110); lactation (*n* = 10); “infant formula” (*n* = 17); “baby formula” (*n* = 3); “infant feeding” (*n* = 3); “infant nutrition” (*n* = 1); presidential documents; breast*(*n* = 18); lactation (*n* = 1); “infant formula” (*n* = 1); “baby formula” (*n* = 0); “infant feeding” (*n* = 0); “infant nutrition” (*n* = 0).

The preliminary search for all federal-level infant feeding policies in the U.S. was carried out using the key search terms of “baby formula,” “breast^*^,” “infant feeding,” “infant formula,” “infant nutrition,” and “lactation.” The search strategy is presented in Figure 1. Two researchers separately scanned each of the full-text records that the search yielded and identified policies for inclusion by ensuring that the policies related to human infant feeding (PH, TK). Any disputes on inclusion were resolved in consultation with a third researcher (SM). After the preliminary screening, two researchers used the Westlaw Edge legal research database to review the policy language in each of the screened records a second time to verify that the policy change occurred within the designated time frame of the search and that the change was substantive (42). Policy change was determined by checking if the policy was new or substantively modified during the study window. Modifications were assessed by comparing the most recent language against previous versions of the policy in the history and credits section of Westlaw Edge for each record using the key search terms. A change was deemed substantive if the policy language differed from previous versions and was relevant to infant feeding. Amendments involving only nomenclature changes were not included. Any discrepancies were resolved in consultation between two researchers (PH, SM).

Infant feeding policy changes that met inclusion criteria were put in a spreadsheet organized by U.S. policy citation, which describes the policy by title, type of policy (e.g., legislation, regulation, presidential document) and section designation. The common names of the policy, date of policy change, whether the policy was new or amended, the infant feeding topic areas addressed in the policy changed, and whether those topic areas aligned with GBC and/or non-GBC policy areas were recorded.

### 2.3 Policy changes compared against GBC policy recommendations

Two researchers (PH, SM) iteratively compared and sorted the identified policy changes into topic areas matching GBC policy priorities (21, 23). U.S. infant feeding policy changes that fell outside of GBC priorities were coded and sorted by the two researchers into non-GBC categories by topic area (e.g., lactation in public or infant formula supply) that were created inductively and informed by a review of the infant feeding policy literature. Any discrepancies were resolved through discussion by the two researchers.

## 3 Results

**Figure 2:**
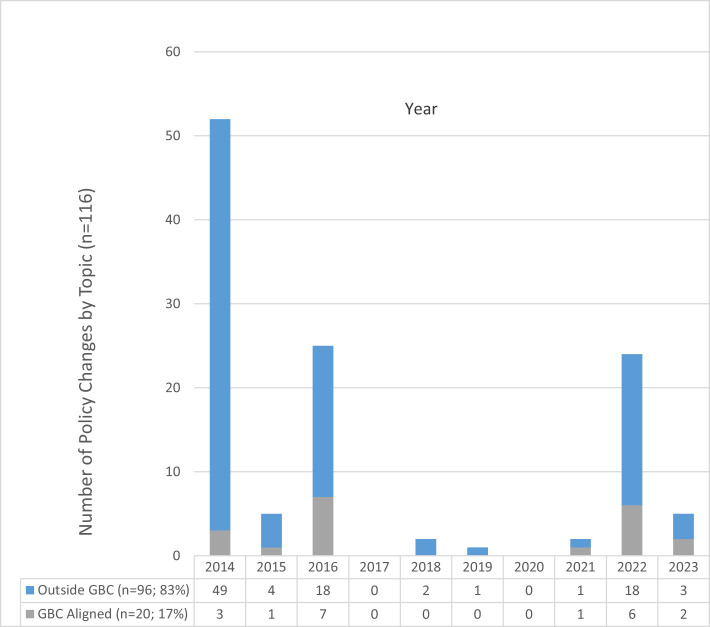
Chart of U.S. policy changes 2014 through 2023 by topic aligned or not aligned with Global Breastfeeding Collective policy priorities by year.

Fifty-seven new or substantively modified U.S. federal infant feeding policies were identified (Table 3). Twenty-nine of the 57 policies (51%) addressed more than one infant feeding policy topic area. Seventeen of the 57 policies (30%) aligned with at least one of the seven GBC policy areas−15 with a single policy priority, one with two policy topic priorities, and one with three GBC priorities (Table 2). In total, the 57 new or substantively changed policies covered 116 topics coded into GBC and non-GBC policy areas; 10 of the 57 policies (18%) addressed both GBC and non-GBC policy areas. Of the 116 topics addressed, 20 (17%) aligned with the seven GBC policy priorities and 96 (83%) topics were outside of GBC policy priorities (Figure 2; Table 3).

**Table 2:** U.S. federal infant feeding policy changes and topics 2014–2023 by GBC policy priorities.

**Common policy name or description**	**Citations of US federal infant feeding policy changes with GBC policy area (*n =* 17)**	**Date of policy change**	**Policy area 2**	**Policy area 3**	**Policy area 4**	**Policy area 5**	**Policy area 6**	**Policy area 7**	**Policy area 8**
			**Marketing infant formula**	**Family leave and/or workplace breastfeeding**	**Hospital**	**Skilled breastfeeding counseling access**	**Strengthen links between health facilities and communities**	**Breastfeeding monitoring**	**IYCF support in emergencies**
Enhancing Flexibilities and Work-life Programs	79 FR 36625	6/27/14							
Procedures Child Development Programs military	32 CFR §79.6	11/16/14							
Medical care for spouses and children contracts (military)	10 USC §1079	12/19/14							
Breastfeeding Policy for the Department of the Army^†^	10 USC Ch. 733	11/25/15							
Requirements for lunches and afterschool snacks	7 CFR §210.10	6/24/16							
Discrimination: pregnancy, childbirth, related conditions	41 CFR §60-20.5	8/15/16							
Child nutrition Head Start	45 CFR §1302.44	9/6/16							
Records and reports WIC	7 CFR §246.25	9/28/16							
Discrimination prohibited based on pregnancy (and lactation)	29 CFR §38.8	12/7/16							
Administration WIC	7 CFR §246.12	12/28/16							
Military Construction: Private nursing and lactation spaces^†^	10 USC §2802	12/27/21							
Access to Baby Formula Act of 2022	42 USC §1786	5/21/22							
Global Malnutrition and Prevention Act of 2021^†^	22 USC §9301	10/19/22							
Installation of audio and video recording devices	49 USC §20168	12/29/22							
PUMP for Nursing Mothers Act	29 USC §218d	12/29/22							
Infant formula and authorized foods cost containment	7CFR §246.16a	12/14/23							
Education and training opportunities for military spouses	10 USC §1784a	12/22/23							
**Total number of topics by GBC policy priorities (*****n** **=*** **20)**			**0**	**8**	**0**	**1**	**4**	**5**	**2**

CFR, Code of Federal Regulation; FR, Federal Record; GBC, Global Breastfeeding Collective; IYCF, Infant and Young Child Feeding; PUMP, Providing Urgent Maternal Protections for Nursing Mothers; USC, United States Code Legislation; WIC U.S. Special Supplemental Program for Women, Infants, and Children.

† Attached to the legislation there was a statutory note related to the infant feeding policy change; Total number of topics by GBC policy areas identified will exceed the 17 citations because an individual legislation, regulation, and/or presidential document can include multiple policy topic areas.

The light grey on the left indicates that the policy change aligned with GBC policy priorities. The darker grey on the right indicates that the policy change did not align with the GBC policy priorities.

**Table 3:** U.S. federal infant feeding policy changes 2014–2023.

Common policy name or description	Policy citation (*n* = 57)	Date of policy change	New or amendment	Infant feeding policy topics aligned with GBC scorecard policy areas							Infant feeding policy topics outside of GBC scorecard policy areas													
				Marketing IF	Family leave and/or workplace breast feeding	Hospital policies	Training skilled BF counselling	BF counseling community	BF monitoring	IYCF in emergencies	BF in public	BF supplies	BF advocacy or education	Research priorities	IF labeling	IF nutrient adequacy	IF manufacturing	IF supply	IF monitoring	Electronic benefit transfer	Transport liquids for infants on airplanes	Infant feeding in child-care settings	Prescription drug labeling	New office of critical food in ^‡^CFSAN report/educate IF
Supplemental foods requirements WIC	7 CFR §246.10	5/5/14	A																					
Controls to prevent adulteration by workers	21 CFR §106.10	6/10/14	N																					
Controls to prevent adulteration caused by facilities	21 CFR §106.20	6/10/14	A																					
Controls to prevent adulteration caused by equipment or utensils	21 CFR §106.30	6/10/14	A																					
Controls to prevent adulteration caused by automatic equipment	21 CFR §106.35	6/10/14	N																					
Controls to prevent adulteration caused by ingredients	21 CFR §106.40	6/10/14	N																					
Controls to prevent adulteration during manufacturing	21 CFR §106.50	6/10/14	N																					
Controls to prevent adulteration from microorganisms	21 CFR §106.55	6/10/14	N																					
Controls to prevent adulteration during packaging and labeling	21 CFR §106.60	6/10/14	N																					
Controls on the release of finished infant formula	21 CFR §106.70	6/10/14	N																					
Traceability of infant formula	21 CFR §106.80	6/10/14	N																					
Audits of current good manufacturing practice	21 CFR §106.90	6/10/14	A																					
General quality control infant formula	21 CFR §106.91	6/10/14	N																					
Audits of quality control procedures infant formula	21 CFR §106.92	6/10/14	N																					
Audit plans and procedures infant formulas	21 CFR §106.94	6/10/14	N																					
Requirements for quality factors for infant formulas	21 CFR §106.96	6/10/14	N																					
New infant formula registration	21 CFR §106.110	6/10/14	N																					
New infant formula submission	21 CFR §106.120	6/10/14	N																					
Quality assurances for infant formulas	21 CFR §106.121	6/10/14	N																					
Verification submission infant formula	21 CFR §106.130	6/10/14	N																					
Submission change in infant formula that may adulterate the product	21 CFR §106.140	6/10/14	N																					
Notification of adulterated or misbranded infant formula	21 CFR §106.150	6/10/14	A																					
Enhancing Flexibilities and Work-life Programs^‡^	79 FR 36625	6/27/14	N																					
Notification requirements infant formula recall	21 CFR §107.240	7/10/14	A																					
Procedures Child Development Programs military	32 CFR §79.6	11/16/14	A																					
Medical care for spouses and children contracts	10 USC §1079	12/19/14	A																					
Specific requirements labeling for human prescription drugs	21 CFR §201.57	6/30/15	A																					
Nutrient specifications infant formula	21 CFR §107.100	10/13/15	A																					
Records infant formula manufacturing	21 CFR §106.100	11/16/15	A																					
Breastfeeding Policy for the Department of the Army^†^	10 USC Ch. 733	11/25/15	N																					
Plan of operations Food and Nutrition Services	7 CFR §272.2	3/31/16	A																					
Nutrient information Infant formula label	21 CFR §107.10	6/22/16	A																					
Requirements for lunches and afterschool snacks	7 CFR §210.10	6/24/16	A																					
Discrimination on the basis of pregnancy, childbirth, or related conditions	41 CFR §60-20.5	8/15/16	N																					
Child nutrition Head Start	45 CFR §1302.44	9/6/16	N																					
Family support services for health, nutrition, and mental health (HHS)	45 CFR §1302.46	9/6/16	N																					
Prenatal and postpartum information, education, and services (HHS)	45 CFR §1302.81	9/6/16	N																					
Records and reports WIC	7 CFR §246.25	9/28/16	A																					
Requirements for meals Child Nutrition Programs	7 CFR §226.20	11/1/16	A																					
Discrimination prohibited based on pregnancy (and lactation)	29 CFR §38.8	12/7/16	N																					
Inclusion of women and minorities in clinical research: Task Force^†^ Pregnancy and Lactation	42 USC §289a-2	12/13/16	A																					
Bottles and Breastfeeding Equipment Screening Act	49 USC §44901	12/16/16	A																					
Administration WIC	7 CFR §246.12	12/28/16	A																					
Airport operations (Mother's rooms)	49 USC §47107	10/5/18	A																					
Terminal development costs (Lactation areas)	49 USC §47119	10/5/18	A																					
Fairness for Breastfeeding Mothers Act	40 USC §3318	7/25/19	N																					
Military Construction: Private nursing and lactation spaces^†^	10 USC §2802	12/27/21	A																					
Access to Baby Formula Act of 2022	42 USC §1786	5/21/22	A																					
Delegating Authority Under the Defense Production Act; Infant Formula	87 FR 31357	5/24/22	N																					
Bulk Infant Formula to Retail Shelves Act	H.R. 8892	10/10/22	A																					
Global Malnutrition and Prevention Act of 2021^†^	22 USC §9301	10/19/22	A																					
Installation of Audio and Video Recording Devices	49 USC §20168	12/29/22	N																					
Infant formulas	21 USC §350a	12/29/22	A																					
Protecting Infants and improving formula supply	21 USC §350a-1	12/29/22	N																					
PUMP for Nursing Mothers Act	29 USC §218d	12/29/22	N																					
Infant formula and authorized foods cost containment	7 CFR §246.16a	12/14/23	A																					
Education and training opportunities for military spouses	10 USC §1784a	12/22/23	A																					
Total number breastfeeding policy changes by policy area	116		*A* = 28, *N* = 29	0	8	0	1	4	5	2	6	2	9	1	7	11	27	10	14	1	1	4	1	2

N, New Policy; A, Amended Policy; CFR, Code of Federal Regulation; FR, Federal Record for Presidential Documents; USC, United States Code Legislation; BF, Breastfeeding; IF, Infant Formula; IYCF, Infant and Young Child Feeding;

† Attached to the legislation there was a statutory note related to the infant feeding policy change; Total number of GBC policy areas identified will exceed the 57 policy citations because an individual legislation, regulation, and/or presidential document can include multiple policy areas.

‡ Center for Food Safety and Applied Nutrition in the FDA.

The light grey on the left indicates that the policy change aligned with GBC policy priorities. The darker grey on the right indicates that the policy change did not align with the GBC policy priorities.

### 3.1 Policy changes aligned with GBC policy priorities

No policies adopted or substantively modified during the study window aligned with the infant formula marketing and hospital-level breastfeeding GBC recommendations. Eight policies addressed unpaid family leave and/or workplace accommodations for breastfeeding. Examples were the 2014 Enhancing Flexibilities and Work-Life Programs presidential memo, which called for private places to breastfeed and unpaid leave time to enhance work-life balance in the workforce, and the 2022 Providing Urgent Maternal Protections for Nursing Mothers (PUMP) Act, which mandated workplace lactation accommodations. Other policies provided increased privacy for breastfeeding military service members and transport workers, seeking to minimize lactation-based discrimination at work.

One policy met the GBC's aim of improving access to skilled breastfeeding counseling, by providing training for military spouses to be certified as doulas or Internationally Board-Certified Lactation Consultants. Four policies covered community-level breastfeeding counseling and/or referrals to counseling. Five policies addressed breastfeeding monitoring.

Two policies aligned with infant and young child support in emergencies. The first was the 2022 Access to Baby Formula Act, an amendment to WIC legislation, which waives administrative procedures for infant formula during U.S. emergencies. The Global Malnutrition Prevention Act (2022), which supported breastfeeding as part of U.S. foreign assistance development aid, also aligned with infant and young child support in emergencies.

### 3.2 Policy changes not aligned with GBC policy priorities

Forty-nine of the 57 policies included topics that did not align with the GBC policy priorities (Table 3). Infant feeding policy topics outside of the GBC priorities included breastfeeding lactation spaces outside the workplace, breastfeeding supplies, breastfeeding education, breastfeeding research, infant formula labeling, infant formula nutrients, infant formula manufacturing, infant formula supply, infant formula monitoring, electronic benefit transfers, the transport of liquids for infants on airplanes, and prescription drug labeling. Changes to Food and Drug Administration operations to oversee infant formula manufacturing and supplies and inform the public about infant formula were made.

Among the non-GBC changes related to breastfeeding, six policies classified under breastfeeding in public aimed to support breastfeeding outside of the home. The 2019 Fairness for Breastfeeding Mothers Act mandated that lactation rooms be made available to visitors in federal buildings. Other policies required providing lactation spaces in airports, military posts, and in child nutrition program sites, such as Head Start. Two policies supported access to breastfeeding supplies (e.g., breast pumps and/or milk storage bags), the 2022 Access to Baby Formula Act amendment to WIC and in 2014 military legislation. Four policies promoted breastfeeding and/or standards governing infant feeding, including procedures to properly store and handle breast milk in child-care programs. Nine policies included breastfeeding education. One modified policy promoted clinical research with protections for pregnant and lactating women.

Policies addressing the manufacturing of infant formula were the most frequent. Fourteen addressed the monitoring of infant formula, including quality control in manufacturing. Separate policies related to infant formula labeling and nutrients in infant formula. None of the policies identified included language on health claims about infant formula, which is recommended in Article 9 of the Code.

Ten policies included changes addressing infant formula supply; six of these 10 were put in place after the 2022 shortage. Those six policies included rules governing administration of the WIC program through the 2022 Access to Baby Formula Act; a 2022 presidential memo delegating executive level authority to ensure and control ingredients under the Defense Production Act; the 2022 Bulk Infant Formula to Retail Shelves Act, which waived duties on infant formula base imported into the U.S.; and an amendment to the regulation guiding WIC in 2023. In 2022, existing infant formula legislation was amended, including outlining new requirements for the Secretary of Health and Human Services to report to Congress and actions to undertake in the event of a future shortage, such as waiving import barriers for specialty infant formulas. Also in 2022, a new section of the infant formula legislation, entitled Protecting Infants and Improving Infant Formula Supply, was added. The new section specified Food and Drug Administration reporting and public communication actions and directed the Food and Drug Administration to develop a National Strategy on Infant Formula to protect infant formula, incentivize increased supply, and mitigate future shortages.

Three other policy topics did not align with GBC priorities: Changes to the WIC program were made to expand the use of electronic benefit transfers for the purchasing of infant formula. The “Bottles and Breastfeeding Equipment Screening Act” in 2016 specified exemptions to the “3–1–1 Liquids Rule” restricting the quantity of breastmilk, infant formula, or other liquids for infants allowed on airplanes. Regulatory changes on the labeling of prescription drugs addressed possible transmission of medicine or drugs through breastmilk to infants and when use of a drug is contraindicated during breastfeeding.

## 4 Discussion

The purpose of this study was to explore how changes in U.S. federal infant feeding policies compared to recommendations in the GBC policy priorities. It complements existing state and national assessments of U.S. breastfeeding policy implementation by examining changes across breastfeeding and infant formula policies and alignment with global priorities (52–57). While 17 of the 57 (30%) policies examined aligned with at least one of the GBC policy priorities, the other 70% did not align with any and the majority (49 out of 57, 86%) included changes outside the scope of the GBC priorities. Although U.S. and global progress for exclusive breastfeeding to the age of 6 months is increasing in comparison to past assessments, progress remains short of targets across multiple breastfeeding indicators (4, 22, 58). The lack of alignment between U.S. and GBC policy priorities suggests missed opportunities and challenges to promote, protect, and support breastfeeding in the U.S., which are consequential because misaligned policies can hinder the achievement of intended outcomes (59, 60). Furthermore, the GBC reported in 2025 that among 106 countries, 75 countries, including the U.S., reported increases in breastfeeding rates since 2017, but 29 countries reported decreases in breastfeeding (22).

Increasing funding to raise breastfeeding rates from birth to 2 years of age is an important GBC priority that was not compared against U.S. federal spending in this study. Assessment of funding was outside the scope and U.S. federal breastfeeding appropriations to agencies and programs is tracked by the United States Breastfeeding Collaborative, a breastfeeding support coalition (61). Modeling estimates of the costs of not breastfeeding in the U.S. document racial/ethnic breastfeeding disparities and that substantial health care costs could be prevented by improved breastfeeding rates (7, 62). A 2024 review concluded that total costs of not breastfeeding were typically calculated to be greater than approximately U.S.$100 billion in the U.S. and U.S.$300 billion globally each year, despite a diverse range of approaches and methods used (62). However, funding estimates to implement breastfeeding strategies vary and may be conservative, in part because the costs focused on health care approaches (e.g., lactation counseling) and other costs such as maternal time and/or social policies (e.g., paid leave) to protect breastfeeding were not adequately incorporated (62, 63).

Moreover, the GBC funding indicator is limited to the percentage of countries achieving a target of at least $5 USD per birth based on donor funding, a metric based on recommendations from the World Health Assembly to reach global exclusive breastfeeding targets (22). According to the 2024 GBC Scorecard report, which highlighted the need for accelerated government and donor commitments for breastfeeding, only 4% of countries globally met the GBC funding metric and already insufficient donor breastfeeding development aid contributions were dropping (22). No U.S. data was provided on the GBC funding indicator in the online GBC Scorecard (23). To help meet the GBC call to accelerate in-country and foreign-assistance donor commitments for breastfeeding worldwide, including medium and high-income countries, harmonization with and additional measures that assess progress toward funding targets across a range of GBC policy priorities, including from the Nutrition Accountability Framework of the Global Nutrition Report that monitors donor commitments (58), is warranted.

No policy changes identified in this study related to the marketing of infant formula. In the absence of Code implementation in the U.S., policies allow for the unrestrained marketing of infant formula and industry self-regulation, impeding breastfeeding and beneficial maternal, infant, and child health outcomes (3, 64–66). Researchers have identified federal policy actions that could be enacted across multiple agencies and levels to protect against deceptive infant formula marketing and health claims to align U.S. policies more closely with the Code, without formal endorsement (64, 67). Examples include ensuring that FDA infant formula labeling regulations follow those in the Code, mandating that infant formula manufacturers with WIC contracts adhere to provisions in the Code, and disallowing the distribution of free infant formula in hospital settings (17, 64, 67). Such policy changes were not identified in this study, but represent a future opportunities for research and policy.

Laws protecting workplace breastfeeding, including the provision of break times and paid maternity leave are associated with improved breastfeeding initiation and increased breastfeeding at 6 months (53, 68–70). The eight breastfeeding and work accommodation policies identified in this study suggest progress in closing the gap between U.S. and global policy priorities related to leave, breaktimes, lactation facilities, and privacy protections (23, 71). However, the U.S. policy of 12 weeks unpaid leave time falls far short on the paid family leave target in the GBC scorecard of 18 weeks of paid maternity leave, which was informed by International Labor Organization recommendations (and additionally fall short of 12-week paid family and medical leave recommendations endorsed by the U.S. Breastfeeding Committee) (23, 72, 73). This is consistent with other studies finding that the U.S. lacks comparable federal family leave policies and workplace breastfeeding protections to other high- and-middle-income countries (56, 69, 72, 74). The U.S. falls behind the number of weeks of leave benefits that are legislated in countries including the United Kingdom (52 weeks of which 39 weeks are paid), every country in Europe and Central Asia (ranging between 14 and 58 weeks), and either behind or on par with countries in the Americas (e.g., Canada has 17 weeks of leave; Uruguay 14 weeks, Jamaica and Mexico each have 12 weeks) (71).

The only policy change that specifically referenced unpaid leave policies and included language about breastfeeding support was the 2014 presidential memo to enhance workplace flexibilities and balance for families. While the 2022 PUMP Act extended breastfeeding privacy and the provision of “reasonable” break periods to a greater range of employee types, including home care workers, drivers, teachers, nurses, and agriculture workers, the PUMP Act does not require paid breaks and does not apply to all employee types (75). This highlights inequalities and persistent policy gaps in U.S. breastfeeding support.

No policy changes related to infant feeding in hospitals or supported the GBC aim to “Implement the Ten Steps to successful breastfeeding in maternity facilities”. According to the CDC, 29% of U.S. live births occur in Baby-Friendly designated hospitals, which falls short of GBC indicator target that >50% of births occur in Baby-Friendly facilities (22, 76). In comparison, the GBC reports from country-level data that none (0%) of the delivery facilities in France and 43% of facilities in Mexico are reported as Baby-Friendly, which raises country level considerations (22). In the U.S., unique factors including the absence of a nationalized health system and the fact that hospital policies are regulated and monitored at the state level may account for not meeting the GBC Ten Steps implementation goal. A 2023 review of Baby-Friendly Hospital Initiative implementation in Mexico found uneven monitoring of Baby-Friendly Hospitals and monitoring of the Ten Steps in facilities (77). Overall, following previous recommendations, these findings highlight the importance of considering country specific contexts and need for timely, publicly available, and transparent data to assess progress, enhance comparability, and evaluate possible replicability of approaches across different settings (14, 56).

Only one policy corresponded to the GBC skilled breastfeeding counseling policy, which was the 2023 amendment to military law aimed to support military spouses to become Internationally Board-Certified Lactation Consultants or doulas, indicating an opportunity for increased training and skill development. Policies that provided community-level lactation counseling included 2016 and 2022 WIC policy changes, 2014 modifications to medical benefits for military families, and 2016 lactation counseling referral changes in Head Start program services. The WIC policy changes were consistent with findings in a review by Anstey et al. (2016) that documented the redoubling of efforts by WIC to improve and expand breastfeeding counseling and address breastfeeding inequalities following the 2011 U.S. Surgeon General's Call to Action to Support Breastfeeding (5, 74). Increasing access to lactation counseling in military benefits likely reflected the influence of the 2014 TRICARE Moms Improvement Act, which was enacted to extend Affordable Care Act breastfeeding counseling and breastfeeding supplies to military members and families (78). The TRICARE program provides benefits to personnel in the U.S. Armed Forces, military retirees, and their dependents.

The GBC policy priority of infant and young child feeding support in emergencies, such as public health emergencies or disasters caused by natural catastrophes, is an overlooked but emerging U.S. policy priority (54, 72). Breastmilk is the safest source of nutrition for infants during emergencies, as infant formula supplies can be disrupted and infant formula donations can be disorganized (72, 79). The 2022 Access to Baby Formula Act included new language waiving administrative procedures during U.S. emergencies or disasters for infant formula, but breastfeeding support was not included, indicating a policy gap to promote and safeguard breastfeeding during emergencies.

Breastfeeding-forward policy changes outside of the scope of GBC policy priorities that could contribute to the achievement of breastfeeding objectives in the U.S., and possibly in other countries, were identified. A promising non-GBC policy that can promote breastfeeding was ensuring lactation spaces outside of the home in work and public spaces. Many mothers have reported feeling embarrassed to breastfeed away from home, are not universally protected from being asked to leave public spaces when lactating, and need support to breastfeed outside of the home (5, 52, 56, 74). Specific to public spaces, six policies were put in place to support breastfeeding outside of the home, including a mandate to provide lactation spaces in airports and in child care program settings. While no U.S. federal policy allows breastfeeding in all private and public locations, some local and state level policies do (52, 56).

Increasing access to breastfeeding supplies is a non-GBC policy with the potential to support breastfeeding. Two changes included language about the provision of breastfeeding supplies to improve breastfeeding outcomes. Breast pumps have been widely used to initiate and maintain breastfeeding (80). Nardella et al. (81) found that breast pump use among mothers enrolled in Medicaid was associated with an average of over 20 more weeks of breastfeeding compared to mothers not using breast pumps, with the strongest associations found among Black and Native American respondents. This suggests a potential for policies that increase breastfeeding supplies to minimize breastfeeding disparities.

This study has limitations. It focused exclusively on U.S. federal-level policy change to assess national attention to infant feeding from policymakers. While the authors cast a wide net to capture policy change across a range of infant feeding topics across GBC and non-GBC topics to detect patterns and interpret policymaker attention, there are gaps in the comprehensiveness and depth in the results. Existing federal policies that were in place but did not change within the 10-year period studied were excluded from the study design. One example is the Patient Protection and Affordable Care Act (ACA) of 2010, which includes prenatal health and workplace protections (57, 72). Formal amendments have not been made to the ACA law, and any changes in rules guiding the implementation of the ACA would have occurred at levels not included in this study. The findings in this study complement but do not replace the findings in other studies assessing the implementation or impacts of topic-specific infant feeding policies (52–54, 56, 57). Future studies could include static infant feeding policies that have been in effect with policy changes for a more complete assessment. In addition, while the focus on federal policy change allowed for an assessment of federal policy priorities, it did not account for state level changes Infant feeding laws have been shown to be variable across different states (52). In agreement with past recommendations, it is important to assess infant feeding policy implementation and changes at the state, global and other levels to determine policy coherence across multiple levels (52, 53, 59, 60, 64).

An additional limitation is that the policies included in the study were each given the same weight when they vary in scope, enforcement mechanisms, and potential for public health impact. For example, passage of the 2022 PUMP for Nursing Mothers Act (29 USC §218d)—an amendment to the 2010 Break Time for Nursing Mothers law in the Fair Labor Standards Act, which made important rule changes expanding workplace accommodations to pump breastmilk—is more significant and broader than a specific rule change in 2014 to enhance the traceability of infant formula (21 CFR §106.80). This comparison demonstrates the challenges of equating the weight the policy changes but also demonstrates the range of U.S. infant feeding policies and the stated commitment of U.S. policy makers to specific topics in infant feeding.

The GBC aim is to increase the adoption, implementation and enforcement of recommended policies to protect, promote and support breastfeeding worldwide (13). The emphasis of this study was on the adoption of U.S. infant feeding policies, by focusing on the identification of policy changes at the federal level over a 10 year period. In line with other studies calling for increased investment in implementation research and evaluation (14, 56, 77), future research should explore in more depth the implementation, enforcement, scope and effectiveness of U.S. infant feeding policies, over more time and at multiple levels to identify patterns, including when there are changes in political priorities.

Future assessment of how U.S. policies align with public health nutrition recommendations in both breastfeeding and infant formula feeding domains is needed. Given the frequent use of infant formula in the U.S., and growing transition to infant formula globally in infant diets (26, 27), there is a justification to explore breastfeeding and infant formula policies together and more comprehensively to assess policy activity, political considerations, as well as determinants and impacts using systematic and robust qualitative and quantitative methods that minimize potential bias and allow for greater comparability over time. Building on past recommendations, future research should highlight which populations benefit the most (and least) from infant feeding policies and consider implications within the global food system and for GBC adaptations (35, 39, 64, 70). To support caregivers working outside of the home in the U.S. and in other countries when appropriate, research that explores and compares the applicability of promising policies that are not GBC priorities but with the potential to support breastfeeding, such as increasing access to breastfeeding supplies, and the impacts of those policies, in various settings should be carried out.

## Data Availability

The original contributions presented in the study are included in the article/supplementary material, further inquiries can be directed to the corresponding author.
